# Comparing the contribution of each clinical indicator in predictive models trained on 980 subacute stroke patients: a retrospective study

**DOI:** 10.1038/s41598-023-39475-x

**Published:** 2023-07-29

**Authors:** Yuta Miyazaki, Michiyuki Kawakami, Kunitsugu Kondo, Masahiro Tsujikawa, Kaoru Honaga, Kanjiro Suzuki, Tetsuya Tsuji

**Affiliations:** 1 Department of Rehabilitation Medicine, Tokyo Bay Rehabilitation Hospital, Chiba, Japan; 2 Department of Rehabilitation Medicine, Keio University School of Medicine, 35 Shinanomachi, Tokyo, Shinjuku-ku 160-8582 Japan; 3 Department of Physical Rehabilitation, National Center of Neurology and Psychiatry, National Center Hospital, Tokyo, Japan; 4 Department of Rehabilitation Medicine, Juntendo University Graduate School of Medicine, Tokyo, Japan; 5 Department of Rehabilitation Medicine, Waseda Clinic, Miyazaki, Japan

**Keywords:** Predictive markers, Prognostic markers, Neurology, Outcomes research

## Abstract

Post-stroke disability affects patients’ lifestyles after discharge, and it is essential to predict functional recovery early in hospitalization to allow time for appropriate decisions. Previous studies reported important clinical indicators, but only a few clinical indicators were analyzed due to insufficient numbers of cases. Although review articles can exhaustively identify many prognostic factors, it remains impossible to compare the contribution of each predictor. This study aimed to determine which clinical indicators contribute more to predicting the functional independence measure (FIM) at discharge by comparing standardized coefficients. In this study, 980 participants were enrolled to build predictive models with 32 clinical indicators, including the stroke impairment assessment set (SIAS). Trunk function had the most significant standardized coefficient of 0.221. The predictive models also identified easy FIM sub-items, SIAS, and grip strength on the unaffected side as having positive standardized coefficients. As for the predictive accuracy of this model, R^2^ was 0.741. This is the first report that included FIM sub-items separately in post-stroke predictive models with other clinical indicators. Trunk function and easy FIM sub-items were included in the predictive model with larger positive standardized coefficients. This predictive model may predict prognosis with high accuracy, fewer clinical indicators, and less effort to predict.

## Introduction

Stroke is the second leading cause of disability worldwide, and it is estimated that there are more than eight million stroke survivors^1^. Disability is a social problem because it significantly impacts the quality of life of patients with stroke and their caregivers and increases healthcare costs^2^. In addition to the acute phase, rehabilitation in the subacute phase reduces the level of disability and improves activities of daily living (ADL)^3^. Japanese convalescent rehabilitation wards for the subacute phase started in 2000, and they improved the discharge to home rate^4^. Since ADL at discharge affect the lifestyles of both the patients and caregivers after discharge, it is essential to predict functional recovery early in hospitalization to allow time for appropriate decisions to be made^5^.

Previous studies reported post-stroke prognosis models for functional recovery using multiple regression analysis^6^. The functional independence measure (FIM)^7^ and Barthel index (BI)^8,9^ are commonly used as indicators of ADL, and these indicators are widely used for predicting ADL^6^. Previous studies suggested that additional clinical indicators, for example, the stroke impairment assessment set (SIAS)^10^, trunk impairment scale^11,12^, and grip strength on the unaffected side^13^ improved the predictive accuracies for discharge FIM scores. The trunk impairment scale was also a significant predictor for discharge BI^14^. In addition, nutrition is an important predictive factor because the improvement of the geriatric nutritional risk index (GNRI) was included as a predictive factor for predicting FIM^15^. The body mass index (BMI) also affects post-stroke functional outcome^16^. Another study of 31 explanatory variables including FIM sub-items of 241 stroke patients reported that FIM sub-items improved predictive accuracy^17^. However, this study built predictive models without a sufficiently powered sample size and might not have reached the maximum predictive accuracy, because at least 20 subjects are needed per explanatory variable to build a predictive model using multiple linear regression analysis^18^. Therefore, most previous studies have not exhaustively compared each clinical indicator. In addition, systematic review articles about post-stroke prognosis predicting FIM or BI scores at discharge showed that functional level (total scores of FIM or BI, sub-items were not mentioned), admission stroke severity such as the National Institutes of Health Stroke Scale (NIHSS), post-stroke deficit including impulsivity, neglect, dysphasia, mini-mental state exam (MMSE) scores, and presence of hemiparesis, stroke-related information (such as previous stroke), and age were significant predictors^6^. In contrast, other clinical indicators were controversial^6^. Although review articles can exhaustively extract many prognostic factors from previous studies, it remains impossible to compare the contribution rate of each predictor because of differences in the data analyzed.

In this study, the aim was to compare the contribution rates of each clinical indicator for predicting FIM at discharge. We hypothesized that the contribution rates of these predictors are directly evaluated by including them within a single predictive model. The predictive model was built with enough participants to achieve this goal. Stepwise multiple regression analysis could be performed with clinical indicators such as age, motor functions on both affected and unaffected sides, and nutrition, in addition to all of the FIM sub-items instead of total FIM scores, because a large enough sample was enrolled (980 participants) to reach the maximum predictive accuracy with 32 explanatory variables. The advantage of stepwise regression analysis is that it identifies explanatory variables from the clinical indicators, reducing the amount of calculation^19^.

## Methods

### Study design and participants

The present study was a retrospective, single-center study. The Ethics Committee of Tokyo Bay Rehabilitation Hospital (267-4) approved this study. Informed consent was obtained by provision of an opt-out option on the Tokyo Bay Rehabilitation Hospital’s website to exclude people refusing participation. The study was conducted according to the principles of the Declaration of Helsinki^20^.

**Figure 1:**
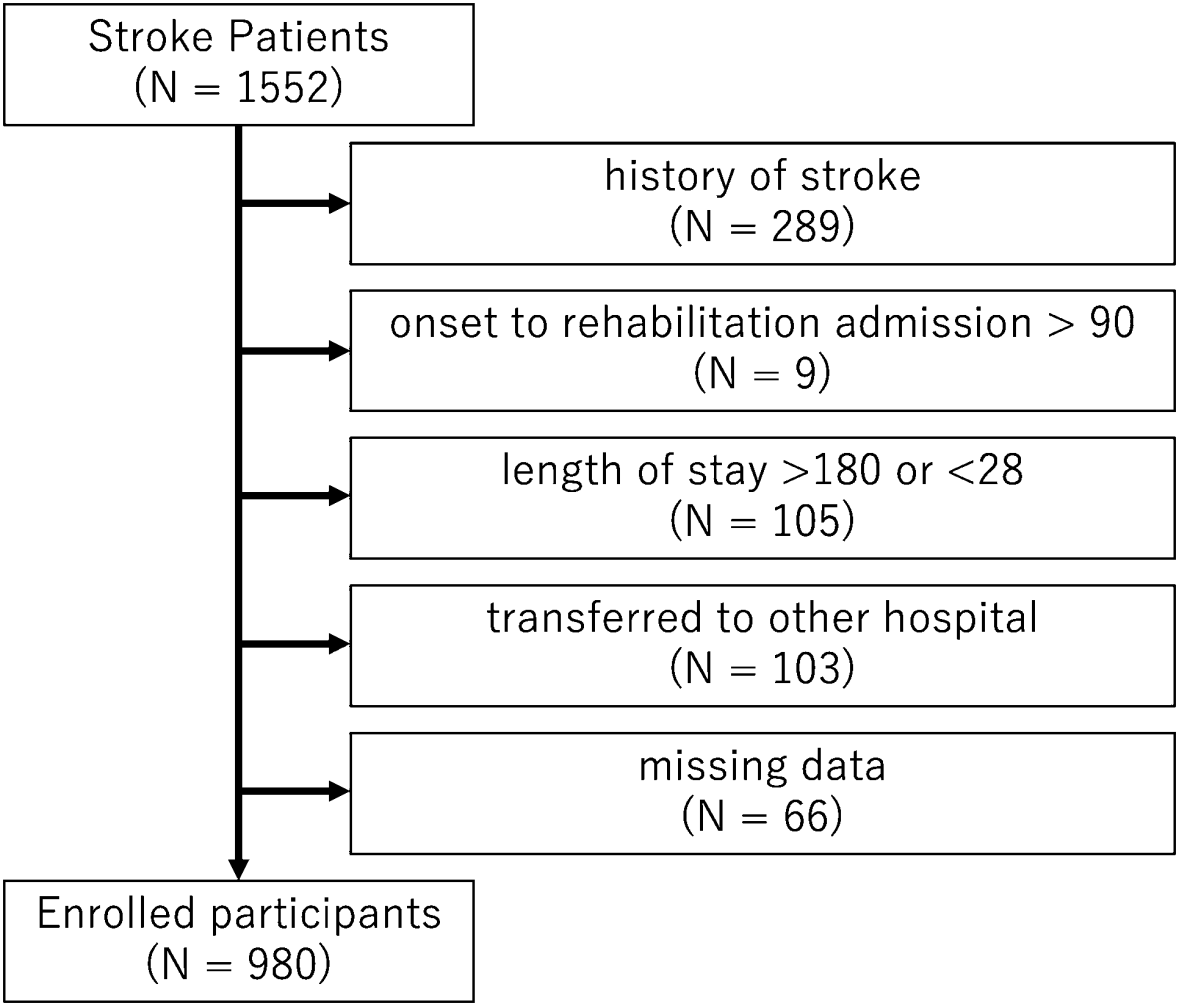
Exclusion criteria.

Between March 2015 and September 2019, 1552 subacute stroke patients were admitted to Tokyo Bay Rehabilitation Hospital, one of the rehabilitation hospitals in which stroke patients receive intensive rehabilitation after acute treatment. The exclusion criteria were (1) history of stroke, (2) onset to rehabilitation hospital admission greater than 90 days, (3) length of stay at our hospital less than 28 days or more than 180 days, and (4) transfer to other hospitals. Missing values in the database were supplemented by referring to the discharge summary. Cases in which missing values could not be completed by referring to the summary (Fig. 1) were excluded. Finally, 980 eligible participants were enrolled after applying the exclusion criteria. All participants in this study underwent approximately 3 h of subacute rehabilitation therapy per day during hospitalization.

### Clinical indicators and data acquisition

Participants’ clinical data were gathered from electronic health records at Tokyo Bay Rehabilitation Hospital, including age, sex, the duration of time between onset and admission (days since onset), FIM sub-items, SIAS sub-items, bilateral grip strength, albumin, BMI, and GNRI.

The FIM is an ADL measure developed in the United States that consists of 13 motor and five cognitive sub-items, with each item scored from 1 to 7 points^7^. The Japanese version of the FIM was used in the present study^21^.

Score on the SIAS is one of the clinical indicators of paralysis in stroke patients^16^. The severity of paralysis is assessed using two items regarding the knee-mouth test and finger movements for the upper extremities, three items regarding hip flexion, knee extension, and ankle dorsiflexion for the lower extremities, and the verticality test, which evaluates trunk function^22^. The SIAS for the upper and lower extremities provides a four-level indicator, in which a score of 0 is most severe and patients cannot move their extremities, and a score of 3 is normal. In the verticality test, a score of 0 is given if the patient cannot maintain sitting. A score of 1 is given if the patient can maintain sitting with support from one side. If the patient can maintain a sitting position by verbal instruction, this is scored as 2. Finally, if the patient is capable of independently remaining in a sitting position, this is scored as 3. The quadriceps strength on the unaffected side is also evaluated. In the quadriceps strength test, a score of 0 is given if the patient cannot move the quadriceps. A score of 3 is given if it is normal.

Bilateral grip strength was measured with a grip strength meter. BMI was calculated as weight divided by height squared. The GNRI is a nutritional risk index calculated using the following formula^23^:

$${\text{GNRI }} = { 14}.{89 } \times {\text{ Alb }}\left( {{\text{g}}/{\text{dL}}} \right) \, + { 41}.{7 } \times {\text{ current body weight}}/{\text{ideal body weight}}{.}$$

Trained nurses evaluated FIM scores at admission and discharge. In addition, therapists and physicians evaluated SIAS scores and bilateral grip strengths. GNRI was calculated using Alb from blood tests performed on admission, height and weight. A total of 32 clinical indicators were used as explanatory variables. The total FIM motor score (FIM-M) can be calculated by summing the 13 motor sub-items.

### Statistical analysis

FIM-M was used as an objective variable in this study. Stepwise regression analysis can automatically select the explanatory variables from a group of clinical indicators for a multiple regression model^19^. Stepwise regression analysis was performed using IBM SPSS Statistics ver. 27 (IBM Corp., Armonk, NY, USA). The stepwise regression analysis calculated unstandardized and standardized coefficients of the explanatory variables. The contribution rates of each clinical indicator can be determined by the standardized coefficients. Multicollinearity was also evaluated. The coefficient of determination (R^2^) for FIM-M at discharge was also calculated.

For multiple linear regression analysis, it is said that at least 20 subjects are needed per explanatory variable to build predictive models with statistically significant variable coefficients^18^. In this study, 32 explanatory variables were included, resulting in at least 640 participants being needed.

## Results

It was calculated that at least 640 participants were needed to build the predictive model with stepwise regression analysis because 32 explanatory variables were included in this study. After applying the exclusion criteria, 980 eligible participants were enrolled in this study (Table 1).

**Table 1:** Participant data.

	Mean ± SD (min–max), n (%)
Age (years)	68.2 ± 14.6 (13 to 98)
Sex	
Female	419 (42.8%)
Male	561 (57.2%)
Days since stroke onset	33.3 ± 12.2 (10 to 86)
Stroke type	
Ischemic	547 (55.8%)
Cerebral hemorrhage	364 (37.1%)
Subarachnoid hemorrhage	69 (7.1%)
Admission FIM motor score	46.8 ± 21.1 (13 to 91)
Discharge FIM motor score	73.3 ± 19.9 (13 to 91)
FIM motor score gain	26.9 ± 14.7 (− 17 to 72)
Admission FIM cognitive score	23.5 ± 8.2 (5 to 35)
Discharge FIM cognitive score	28.0 ± 7.1 (5 to 35)
Admission FIM total score	70.2 ± 27.4 (18 to 126)
Discharge FIM total score	101.4 ± 25.7 (18 to 126)
Grip strength on the affected side (kg)	11.9 ± 11.6 (0 to 49.4)
Grip strength on the unaffected side (kg)	22.1 ± 10.8 (0 to 52.6)
BMI (kg/m^2^)	22.2 ± 3.7 (13.2 to 39.3)
GNRI	97.9 ± 10.5 (64.9 to 136.9)

*FIM* functional independence measure, *BMI* body mass index, *GNRI* geriatric nutritional risk index.

The clinical indicators using in stepwise multiple regression analysis are shown in Table 2; they included age, days since onset, FIM (bowel management, eating, bed/chair transfer, grooming, bathing, and social interaction), SIAS (the verticality test, knee extension, quadriceps strength on the unaffected side), grip strength on the unaffected side, and BMI.

**Table 2:** Standardized and unstandardized coefficients of clinical indicators for the FIM motor prognosis model.

	Standardized coefficient	Unstandardized coefficient		t	p	VIF
	Beta	B	Std. error			
(Constant)		49.24	3.65	13.49	< 0.001	
SIAS-verticality test	0.221	5.34	0.56	9.58	< 0.001	2.00
FIM-bowel management	0.142	1.25	0.27	4.71	< 0.001	3.42
FIM-eating	0.125	1.36	0.29	4.63	< 0.001	2.76
FIM-bed/chair transfer	0.112	1.15	0.35	3.29	0.001	4.39
FIM-grooming	0.105	1.09	0.37	2.95	0.003	4.74
FIM-social interaction	0.105	1.26	0.27	4.64	< 0.001	1.94
SIAS-knee extension	0.101	1.21	0.30	3.97	< 0.001	2.43
Grip strength on the unaffected side	0.085	0.16	0.04	3.84	< 0.001	1.83
SIAS-quadriceps strength on the unaffected side	0.056	1.21	0.42	2.85	0.005	1.45
BMI	− 0.037	− 0.20	0.10	− 2.07	0.038	1.17
FIM-bathing	− 0.068	− 0.72	0.27	− 2.67	0.008	2.41
Days since onset	− 0.087	− 0.14	0.03	− 5.07	< 0.001	1.11
Age	− 0.189	− 0.26	0.03	− 9.74	< 0.001	1.42

*BMI* body mass index, *FIM* functional independence measure, *MMT* manual muscle test, *SIAS* stroke impairment assessment set, *VIF* variance inflation factor.

Trunk function evaluated by the SIAS-verticality test had the most significant standardized coefficient of 0.221. Therefore, trunk function contributes the most to predicting FIM-M at discharge. The predictive model also included FIM sub-items (bowel management, eating, bed/chair transfer, grooming, and social interaction), knee extension, grip strength on the unaffected side, and quadriceps strength on the unaffected side, with positive contribution coefficients ranging from 0.056 to 0.142.

The results showed that FIM sub-items contributed more to the predictive model than motor functions on both the affected and unaffected sides. The predictive model included only SIAS-knee extension from the motor function items on the affected side. In contrast, BMI, bathing, days since onset, and age had negative coefficients ranging from − 0.189 to − 0.037. In addition, the predictive model did not include GNRI and other FIM and SIAS sub-items. The predictive model in this study did not include paralysis severity except that for knee extension, despite it being a predictive model of ADL at discharge.

As for the predictive accuracy of this model, R^2^ was 0.741 (*p* < 0.001). The level of significance was set at p < 0.05, and the variance inflation factor (VIF) was less than five, without collinearity.

## Discussion

The results of the present study suggested that the SIAS-verticality test, FIM sub-items, SIAS-knee extension, and function of the unaffected upper and lower extremities’ contributed to the predictive model for FIM-M. Previous studies have examined various prognostic factors, but it was not easy to compare the contribution of each predictor in a review article. In addition, FIM sub-items were not mentioned in a review article about predicting stroke patients’ FIM^6^.

### Trunk function

The SIAS-verticality test, for which the beta was 0.221, contributed the most to the predictive model for FIM-M at discharge in the present study. The previous review article reported that trunk function was one of the predictors^6^. Moreover, to the best of our knowledge, no study has predicted FIM-M at discharge by simultaneously using trunk function and FIM sub-items on admission. We had considered that FIM sub-items on admission might have a higher contribution rate to prediction of FIM-M at discharge than those of other indicators because they reflect the same group of impairments. However, the present study suggested that score on the SIAS-verticality test had the best contribution rate. A previous study reported that trunk function is one of the factors affecting post-stroke gait function and ADL^24^. Another study also reported that trunk function, primarily as assessed by static sitting balance, is an important prognostic factor for post-stroke ADL^14^. One possibility is that trunk function may have contributed the most to the FIM prognosis model, since stability in the sitting position is required for most ADL. Therefore, this study suggested that trunk function as evaluated by the SIAS-verticality test could be the most important prognostic factor.

### FIM sub-items

We compared the contribution of each FIM sub-item to the predictive model in the present study. Bowel management, eating, bed/chair transfer, grooming, social interaction, and bathing were adopted in the predictive model. The present study showed that the contribution rates of these FIM sub-items were larger than those of other clinical indicators, except for the SIAS-verticality test. These results suggested that the SIAS-verticality test and the FIM sub-items used in the present prognostic model may be used to predict FIM-M at discharge with good prognostic accuracy in a minimum amount of time. An ADL structural analysis in stroke patients reported that eating, bowel management, grooming, bed/chair transfer, and social interaction were the easy sub-items^25^. The present study included the easy sub-items as predictors with positive coefficients. Participants who are not assessed as independent on easy sub-items at admission are likely to be severely affected, and their FIM-M at discharge could be expected to likely also be low. In contrast, if the participants are not independent on difficult sub-items at admission, it is difficult to determine whether the participants are too severely affected to improve their function or will improve with rehabilitation, making it difficult to predict prognosis. Therefore, we considered that these easy sub-items were selected to predict the severity and potential of functional improvement. On the other hand, only bathing was a prognostic predictor with a negative coefficient. Moreover, bathing is one of the most challenging FIM motor sub-items analyzed by Rasch analysis^25^, and this indicates that patients who are independent in bathing would likely be independent in other FIM-M sub-items. Thus, it is considered that their improvements were small due to the ceiling effect. The negative coefficient for bathing may have been used to reduce the FIM-M at discharge for patients who were independent in bathing at admission.

### Other motor functions

In addition to trunk function and FIM sub-items, the present study included SIAS-knee extension, and function of the unaffected upper and lower extremities’ as prognostic factors. These explanatory factors may have lower priorities for inclusion in the predictive models, because they had lower contributions to the predictive model than the SIAS-verticality test and FIM sub-items. Since knee function has been reported to be an essential factor for improving gait function after stroke^26^, SIAS-knee extension was employed as a predictor in this study. Moreover, another previous study predicting gait independence 3 months after onset reported that a decrease in quadriceps muscle mass on the unaffected side was a predictor^27^. Therefore, quadriceps strength on the unaffected side may influence ADL, and contribute more to predictive models than on the affected side.

The grip strength on the unaffected side also contributed to our predictive model. Grip strength is one of the diagnostic criteria for sarcopenia^28^ and is a prognostic factor for disability and reduced quality of life^29,30^. It is also a predictor of independence in stroke patients^13^. Therefore, grip strength on the unaffected side may contribute to the predictive model by reflecting sarcopenia, consistent with previous studies.

In contrast, the present predictive model did not include upper extremity function on the affected side as a prognostic factor. Since most ADL can be performed independently with only one extremity on the unaffected side, impaired upper extremity function has a lower impact on ADL than do trunk function and upper extremity function on the unaffected side^31^.

### Other explanatory factors

In the present study, age, days since onset, and BMI were included as explanatory factors with negative correlation coefficients. Previous studies reported negative coefficients for age and days since onset^32,33^. The present study also suggested that these factors were risk factors for post stroke functional recovery, and age had the worst contribution to the predictive model. In addition, obesity may associate with a good prognosis with highest FIM efficiency^16^, but another study reported that obesity is still controversial^34^. In the present study, BMI had a negative coefficient. One possibility that the results of Western studies may not be directly applicable or generalizable to the Japanese population because the percentage of individuals with BMI ≥ 30 kg/m^2^ is lower in Japan than in Western populations^35^.

The present results suggest that trunk function may contribute the most to the predictive model of FIM at discharge and paralysis severity. Furthermore, the results also suggest that easy FIM motor sub-items contributed more than motor functions other than trunk function. Upper and lower muscle strength on the unaffected side could contribute more to the predictive model than those on the affected side, except knee function. In summary, trunk function, motor function on the unaffected side, and independence in easy FIM sub-items could be important factors for predicting FIM at discharge. On the other hand, it is difficult to prove causal relationships because this was a retrospective study. Future cohort studies will be needed to establish causal relationships between the clinical indicators and the observed outcomes.

### Coefficient of determination

The coefficient of determination in the present study was 0.741 for FIM-M at discharge. The review article on multiple regression analysis for stroke patients reported a mean coefficient of determination of 0.65 (0.35–0.82) for FIM-M^6^. Therefore, the present predictive accuracy for FIM-M was better than the average of previous studies.

### Study limitations

There were several limitations to this study. First, this was a single-center study. Therefore, the participants would have been a select group from the overall population. A multi-center study is needed to better generalize the findings. Second, the present study was a retrospective study, and there were missing cases, due to, for example, incomplete documentation and clinical indicators. In addition, only clinical indicators that we usually evaluated in daily practice and were reported in previous studies were collected. Therefore, we could not gather other clinical indicators, such as those for psychosocial, environmental, and lifestyle factors. Furthermore, retrospective studies suggest only possible causal relationships between studied factors and the observed outcomes. A future cohort study will be considered to avoid missing cases, include other critical clinical indicators, and establish causal relationships. Third, potential confounders could affect the internal validity of the present results. The VIFs of the clinical indicators selected in this study were less than five, and statistically obvious confounders were not observed. However, one cannot rule out that the associations found were influenced by factors unaccounted for. Therefore, future studies will need to consider additional potential confounders. Fourth, brain imaging, such as computed tomography and magnetic resonance imaging, was not evaluated. Brain images could possibly have provided more information, allowing improvement of predictive accuracy. While use of deep learning techniques could have potentially uncovered much more such information from brain imaging, but this could not be done for the present study due to an insufficient number of brain imaging examinations. Finally, application of machine learning and deep learning techniques to improve predictive accuracies were in addition not considered because they have difficulty with explaining explanatory factors. In future research, machine learning models need to be considered.

## Conclusion

The present research aimed to compare the contribution rate of each clinical indicator for predicting post-stroke functional prognosis. The present study showed that trunk function contributed the most to FIM-M. In addition, easy FIM sub-items contributed more than other clinical indicators except for the SIAS-verticality test. Easy FIM motor sub-items were included in the FIM-M predictive model with positive standardized coefficients. Moreover, quadriceps strength and grip strength on the unaffected side also contributed to predictive models more-so than those on the affected side. This is the first report to include FIM sub-items separately in post-stroke predictive models with other clinical indicators. This predictive model may predict prognosis with high accuracy, fewer clinical indicators, and less effort to predict. This was a retrospective study and had some limitations. For example, it is difficult to rule out the possibility of potential confounders and establish causal relationships. Therefore, it is necessary to consider these limitations in future cohort studies.

## Data Availability

The present research involved human research participant data, which raises ethical issues regarding the protection of personal information, and we did not receive approval from the Ethics Committee to share our data publicly. However, the data are available on reasonable request with permission from the Ethics Committee of the Tokyo Bay Rehabilitation Hospital. Researchers can contact the corresponding author (Michiyuki Kawakami), or the Ethics Committee of the Tokyo Bay Rehabilitation Hospital by e-mail or letter correspondence. The e-mail address of Michiyuki Kawakami is michiyukikawakami@hotmail.com. The e-mail address of the Ethics Committee of the Tokyo Bay Rehabilitation Hospital is shinsakai@wanreha.net. The mailing address of the Ethics Committee in the Tokyo Bay Rehabilitation Hospital is 4-1-1 Yatsu, Narashino, Chiba, Japan.

## Abbreviations

FIM: Functional independence measure
FIM-M: Total FIM motor score
SIAS: Stroke impairment assessment set
ADL: Activities of daily living
BI: Barthel index
GNRI: Geriatric nutritional risk index
BMI: Body mass index
NIHSS: National Institutes of Health Stroke Scale
MMSE: Mini-mental state exam
